# Choosing between lobectomy or segmentectomy: moving beyond the two-centimetre debate

**DOI:** 10.1093/icvts/ivaf113

**Published:** 2025-05-20

**Authors:** Herbert Decaluwé

**Affiliations:** Department of Thoracovascular Surgery, Ziekenhuis Oost-Limburg, ZOL Genk, Genk, Belgium

**Keywords:** NSCLC, segmentectomy, lobectomy, sublobar, tumor size

‘Why would you take out half of a patient’s lung for that lesion?’

This pointed question resurfaces with renewed relevance in the era of high-resolution imaging and recent trials revisiting the balance between oncologic safety and parenchymal preservation. With the publication of landmark randomized studies—JCOG0802 and CALGB 140503—sublobar resections, long practised in selected patients, have now firmly re-entered centre stage, this time backed by robust evidence. As a result, case discussions in multidisciplinary team (MDT) meetings are more nuanced—and more contested—than ever.

These trials redefined the surgical standard for peripheral NSCLC lesions ≤2 cm with ≥50% solid component, showing that segmentectomy is not only non-inferior but may also offer superior overall survival compared to lobectomy [1, 2]. Yet, this survival benefit comes paradoxically without clear improvement in postoperative respiratory function, and with increased loco-regional recurrence. The survival advantage appears driven by non-cancer-related factors, with speculation pointing to better preservation of vascular integrity and options for future interventions.

A critical nuance is that conclusions from JCOG0802 and CALGB 140503 apply to patients eligible for lobectomy but who underwent intentional sublobar resection. In contrast, the approach in functionally compromised patients—those who are medically no candidates for lobectomy—has historically permitted sublobar resection even for larger lesions.

Our current practice reflects a cautious expansion of indications mainly based on Japanese literature. We consider sublobar resections in cases of a consolidation-to-tumour ratio (CTR) ≤0.25 and lesions ≤2 cm. For larger lesions up to ≤3cm (and CTR ≤0.25) we consider segmentectomy based on JCOG1211 [3]. For lesions with CTR >0.25 and ≤2 cm, segmentectomy is offered instead of lobectomy if margins, nodal status and functional considerations allow it. Notably, the recent ESTS expert consensus has raised concern regarding functional outcomes when more than two segments are resected [4].

In this issue, Huang *et al.* evaluate the results of segmentectomy in patients with cIA3 NSCLC (tumours >2 cm but ≤3 cm) compared with patients with cIA1-2 [5]. In this multicentre study of 589 patients, including 111 with cIA3 disease, the authors demonstrate that segmentectomy—when performed in carefully selected patients—yielded comparable recurrence-free survival and perioperative outcomes between cIA3 and cIA1-2 cohorts. Nearly half of the cIA3 tumours were solid lesions.

This study provides much-needed procedure-specific evidence, reinforcing what some thoracic surgeons have cautiously practised: that segmentectomy may be oncologically adequate beyond the 2 cm cut-off, provided selection is meticulous. Still, one must interpret these findings in the context of limitations—namely, a relatively short median follow-up of 24 months and the underpowered nature of subgroup analyses, particularly for centrally located lesions.

A recent European survey found that less than 10% of surveyed surgeons would choose for segmentectomy when the lesion is larger than 2 cm [6]. In our opinion, tumour location is a crucial determinant in surgical planning. In the same survey, only 15% would decide for segmentectomy based on location in the lobe, yet this parameter encompasses margin feasibility, risk of pleural invasion, and overall surgical complexity. Anatomical considerations—especially whether a segmentectomy can achieve a margin greater than the tumour diameter or 2 cm—often dictate whether the oncologic resection will be adequate.

Beyond anatomy, shared decision-making should incorporate patient expectations, functional status, comorbidities, expected tumour biology and future treatment eligibility. Emerging tools may soon enhance and personalize these complex decisions. Artificial intelligence (AI), already showing promise in predicting outcomes of targeted therapies and optimizing radiotherapy plans, could support more nuanced treatment planning in early-stage NSCLC [7]. A concerted effort among surgeons to include high-quality data from both sublobar and lobar resections will be essential to develop robust AI platforms to guide future resection strategies. Additionally, tissue biopsies from navigational bronchoscopy and molecular markers such as circulating tumour cells may play a future role in risk stratification and treatment choice.

Until such platforms are validated and widely adopted, we must rely on robust MDT discussions to navigate the grey zones. The study by Huang *et al.* does not end the debate—it shifts it. From a binary question of lesion size to a broader inquiry into the interplay between biology, anatomy, patient context, and evolving technology.

In lesions ≤3cm, segmentectomy may be ‘enough’—but only when we ask the right questions.
